# Evaluation of a Pilot Program to Prevent the Misuse of Prescribed Opioids Among Health Care Workers: Repeated Measures Survey Study

**DOI:** 10.2196/53665

**Published:** 2024-04-12

**Authors:** Stephen Hebard, GracieLee Weaver, William B Hansen, Scarlett Ruppert

**Affiliations:** 1 Prevention Strategies Greensboro, NC United States; 2 Department of Public Health Education University of North Carolina Greensboro Greensboro, NC United States

**Keywords:** health care workers, opioid misuse, pain management, prescription opioids, prevention, substance abuse, substance use, workers

## Abstract

**Background:**

Overprescription of opioids has led to increased misuse of opioids, resulting in higher rates of overdose. The workplace can play a vital role in an individual’s intentions to misuse prescription opioids with injured workers being prescribed opioids, at a rate 3 times the national average. For example, health care workers are at risk for injuries, opioid dispensing, and diversion. Intervening within a context that may contribute to risks for opioid misuse while targeting individual psychosocial factors may be a useful complement to interventions at policy and prescribing levels.

**Objective:**

This pilot study assessed the effects of a mobile-friendly opioid misuse intervention prototype tailored for health care workers using the preparation phase of a multiphase optimization strategy design.

**Methods:**

A total of 33 health care practitioners participated in the pilot intervention, which included 10 brief web-based lessons aimed at impacting psychosocial measures that underlie opioid misuse. The lesson topics included: addiction beliefs, addiction control, Centers for Disease Control and Prevention guidelines and recommendations, beliefs about patient-provider relationships and communication, control in communicating with providers, beliefs about self-monitoring pain and side effects, control in self-monitoring pain and side effects, diversion and disposal beliefs, diversion and disposal control, and a conclusion lesson. Using a treatment-only design, pretest and posttest surveys were collected. A general linear repeated measures ANOVA was used to assess mean differences from pretest to posttest. Descriptive statistics were used to assess participant feedback about the intervention.

**Results:**

After completing the intervention, participants showed significant mean changes with increases in knowledge of opioids (+0.459; *P*<.001), less favorable attitudes toward opioids (–1.081; *P*=.001), more positive beliefs about communication with providers (+0.205; *P*=.01), more positive beliefs about pain management control (+0.969; *P*<.001), and increased intentions to avoid opioid use (+0.212; *P*=.03). Of the 33 practitioners who completed the program, most felt positive about the information presented, and almost 70% (23/33) agreed or strongly agreed that other workers in the industry should complete a program like this.

**Conclusions:**

While attempts to address the opioid crisis have been made through public health policies and prescribing initiatives, opioid misuse continues to rise. Certain industries place workers at greater risk for injury and opioid dispensing, making interventions that target workers in these industries of particular importance. Results from this pilot study show positive impacts on knowledge, attitudes, and beliefs about communicating with providers and pain management control, as well as intentions to avoid opioid misuse. However, the dropout rate and small sample size are severe limitations, and the results lack generalizability. Results will be used to inform program revisions and future optimization trials, with the intention of providing insight for future intervention development and evaluation of mobile-friendly eHealth interventions for employees.

## Introduction

There is an opioid crisis in the United States [1,2]. This includes not only the morbidity and mortality associated with the illegal sale and use of opioids [3,4] but also the misuse and abuse of prescribed opioids [5-8]. Prescription opioids are often prescribed to address injuries and pain. In the United States, at least 20% of adults reported experiencing pain every day or most days over the past 6 months [9], and 36% of those with chronic pain reported that their pain frequently limits their life and work activities [10]. The overprescription of opioid pain relievers, such as oxycodone and hydrocodone, has led to widespread addiction, and increased demand for illicit opioids like heroin and fentanyl. In 2020, there were an estimated 91,000+ overdose deaths, with a marked increase in deaths due to opioids since 2016 [11]. Interventions are needed to address the growing problems associated with opioid misuse.

A primary response to the opioid crisis has been to call for improved opioid prescribing practices, improved treatment retention, and increased involvement of psychiatrists [12]. Many educational programs related to prescription opioids are designed for physicians, nurses, and other health care providers and focus on the assessment and treatment of opioid use disorder (ie, buprenorphine) or aligning one’s prescription behaviors with the clinical guidelines for opioid use [13-15]. Proposed strategies for addressing misuse, abuse, and addiction often rely on health care providers for program or intervention delivery, often referred to as patient-centered education programs [16-19]. These strategies can benefit from considering individual risk factors such as a history of pain, previous substance use, or mental health challenges, which could inform delivery, implementation, or the focus of intervention [20]. Previous intervention studies on opioid misuse can also inform future prevention efforts.

In many studies that address education about opioids, knowledge of risks and safe practices is the primary outcomes, and increases in knowledge are frequently observed. For example, in a randomized pilot study, greater knowledge gains were found for those who received a 6-minute video on proper usage of opioids compared to those who received standard-care instructions [21]. An educational campaign using an advertising format focused on changing attitudes and beliefs of a young adult target population showed increases in empathy and perceived risk of problems associated with opioid use [22]. A multidisciplinary clinic-based strategy that included the assessment and treatment of veterans diagnosed with unsafe opioid use patterns suggested better opioid management, but only for a small group of patients [23]. Another study that proposed adaptive prescription and consumption monitoring found significant declines in the risk of developing an opioid addiction associated with their smart prescription management intervention [24]. Among the key takeaways from these studies is the potential for affecting opioid use outcomes through increased provider-patient communication, a key component that other studies have also supported [25,26].

Although interventions for patients and providers may target the point of access and use, they may miss opportunities to establish supportive cultures and environments, such as those that could be implemented across worksites. Worksites represent a critical social determinant of prescription opioid misuse. Despite the risks associated with prescription opioid use, injured workers are frequently prescribed opioid pain medications at rates three times the national average [27,28] and almost one-third of injured workers continue to fill their prescriptions 90 days after their injury [29]. As many as 57% of accidental deaths due to opioid overdose occur following work injuries [30]. In addition, prescription opioid misuse costs US employers an estimated US $25-$26 billion each year due to lost productivity, turnover, and premature employee death [28,29]. Though 70% of employers report negative consequences of opioid misuse (eg, absenteeism, poor performance, and turnover), very few employers offer any type of prescription opioid prevention intervention [31] or feel prepared to address opioid-related concerns [32].

Intervening within worksites would allow for reaching a broader audience, implementing primary prevention before treatment of injury or pain, and addressing complex and unique social-behavioral motivators for behaviors related to health outcomes [33]. For example, coworker relationships can influence beliefs and attitudes about behaviors such as the misuse of prescription drugs [34]. In the case of construction workers, nurses, and nursing assistants, peer normalization of job strain, work-related injuries, and working through pain compounds the risk of musculoskeletal injury and the use of prescription opioids for pain [35,36]. While interventions aimed at preventing other substance use disorders have successfully targeted behavioral factors, particularly through web-based digital health technologies [37,38], a one-size-fits-all approach may ignore the unique predictors of prescription opioid misuse and the influence of work on behavior.

Among the strategies that have been proposed to increase the frequency and quality of communication is the development of smartphone apps [39,40]. Evidence-based mHealth interventions have become a popular and effective delivery method for health behavior [41,42] as 85% of Americans own a smartphone [43]. Further, mHealth interventions have demonstrated exceptional feasibility and effectiveness in the prevention of substance use among a variety of populations [44]. mHealth interventions can be more cost-effective (eg, professional staff do not need to be trained or hired to deliver the content and have a broader reach than facilitator-led interventions) [45].

This pilot study examines the use of an mHealth intervention for prescription opioid use prevention, referred to as “WorkWell,” which targets psychosocial outcomes that research suggests may influence intentions to use or misuse opioids, such as knowledge, attitudes, perceived control of managing pain, and perceptions about communicating with providers [21-26,46,47]. WorkWell was designed to be delivered digitally because of the economy, privacy, scalability, and impact that mHealth interventions can have. In addition, brief mHealth interventions like WorkWell are ideal for workforces because (1) they can be accessed anytime, allowing self-paced learning; (2) they can be delivered outside of work hours and therefore do not disrupt safety and productivity during the workday; (3) workers can access them in any setting that provides access to wireless internet or a cellular network; and (4) they can deliver personalized feedback while being standardized to ensure fidelity. This paper presents information about the intervention and study protocols, as well as the preliminary results from the pilot data. This pilot study aimed to assess its short-term impact on targeted psychosocial outcomes, as described below.

## Methods

### Intervention

WorkWell presents information about pain management strategies, how opioids may impact acute and chronic pain, guidelines for use and disposal, the nature of the epidemic, tolerance and addiction, and the risks of overdose. In collaboration with an expert advisory panel with backgrounds in generalized pain and symptom management, prescription opioid use among young adults, and workplace culture, we scripted 10 brief lessons (referred to as touch points for participants). With input from the advisory panel, the following lessons targeted attitudes and beliefs: addiction beliefs, Centers for Disease Control and Prevention guidelines and recommendations, patient-provider relationships and communication, beliefs about self-monitoring pain and side effects, diversion and disposal, and the conclusion lesson. Lessons that targeted perceived control included addiction control, communicating with providers, control with self-monitoring pain and side effects, diversion and disposal, and the conclusion lesson. All lessons included knowledge components, and lessons 4 and 5 addressed aspects of patient-provider communication, a key point highlighted in previous research, as well as the input from our expert advisory panel.

We partnered with an educational design team to develop and narrate each lesson and the web-based platform within which it would be housed. Each lesson was expected to be completed in between 8 and 10 minutes. Lessons included text, animations, still images, and dynamic exercises in which participants indicated their experiences, preferences, or decisions when prompted with a scenario. For example, true or false questions were integrated into knowledge components within lessons, and attitudinal components included opportunities for participants to react or input their own beliefs to trigger tailored responses within the app. In addition to tailored feedback in response to their interactions with the material, participants received in-app industry-specific prescription opioid information and appropriately timed push notifications to prompt use and completion.

The prototype developed for pilot testing was tailored for delivery to health care workers, including images or examples specific to working in the health care industry. Nurses are among the most injured workers, just behind construction workers [9,48,49] and nursing assistants had the highest incidence rate of injury cases that required missed days of work [50]. Further, nurses, nursing assistants, and psychiatric aides are at high risk for musculoskeletal disorders and experience considerable work-related stress and job strain (ie, high psychological workload and low work-related decisional latitude) that challenge effective coping and lead to substance use and other behavioral, physical, and mental health concerns [51]. Prescription drug diversion has been cited as a specific problem among nurses, whose access to prescription opioids presents an additional challenge to misuse prevention [52,53]. Given the broad normalization of pain, injury, and musculoskeletal disorders and the distress associated with the perceived need to prematurely return from injury to work, it is clear that employment within this industry is an important risk factor for prescription opioid misuse.

### Participants

Development of this intervention prototype took place in 2019, leaving recruitment for this pilot project to take place during the height of the COVID-19 pandemic. This not only limited in-person or higher-touch recruitment efforts but also created challenges for recruiting from an already overburdened workforce. We relied on recruitment emails sent out through relevant listserves by partnering with “INFOCUS Marketing,” the National Association of School Nurses, and nursing schools at 2 local universities. We were unable to track how many email addresses were valid across various listserves and were thus unaware of the total number of recruitment emails successfully sent. Although 856 health care workers fully completed the pretest survey, only 47 individuals continued to further participate in the intervention. Of those participating in the intervention, 33 (70%) individuals completed all aspects of the study, including the pretest, intervention, and posttest. Participants ranged in age from 26 to 73 years, with a mean age of 49 years. Most participants were female (30/33, 91%), White (31/33, 94%), and (30/33, 91%) current health care practitioners.

### Measures

Pretest and posttest surveys assessed demographics (age, gender, ethnicity, race, and employment) and the following psychosocial measures: knowledge of opioids (eg, “I know how to safely use an opioid prescription for pain;” 7 items; pretest α=.873; posttest α=.935), attitudes toward opioids (eg, “rate from good to bad: using opioids to control pain is...;” 6 items; pretest α=.736; posttest α=.866), communicating with providers (eg, “rate from not important to very important: communicating with my provider about my opioid prescription is...;” 4 items; pretest α=.702; posttest α=.935), pain management control (eg, “rate from very difficult to very easy: refusing a prescription for opioids when I am in pain would be...;” 8 items; pretest α=.718; posttest α=.758), and intentions about opioid use (eg, “rate from strongly disagree to strongly agree: I do not intend to take prescription opioids for pain management;” 4 items; pretest α=.581; posttest α=.735).

At the posttest, participants provided ratings about the WorkWell program and its appropriateness as an educational intervention. Ratings on a 6-point scale ranging from “strongly disagree” to “strongly agree” were given to four prompts: (1) “The information presented in this program was too basic,” (2) “The information presented in this program was difficult to understand,” (3) “The information presented in this program was so boring that I became distracted,” and (4) “All workers in my industry should complete a program like this.”

### Design, Hypotheses, and Analysis Plan

The pilot study was conducted as a pretest-treatment-posttest design without a control or comparison group. Pretest surveys were administered through Qualtrics (Silver Lake). Once participants had completed pretest surveys, the first touch point would be made accessible within the intervention platform. As each touch point was completed, the participant was given access to the next touch point. Therefore, all participants completed all touch points in the same order. Immediately following the completion of all 10 touch points, a posttest survey was made available, again administered through Qualtrics.

We hypothesized that there would be measurable improvements on all targeted psychosocial measures. We also hypothesized that ratings would reflect a positive evaluation of the intervention. Within-subject pretest and posttest psychosocial measures were compared using a general linear repeated measures analysis of variance. Descriptive statistics were provided for participants’ posttest ratings about the program.

### Ethical Considerations

This study was reviewed and approved by the University of North Carolina Greensboro Institutional Review Board (#20-0090). Recruitment emails included information about the purpose of the study, what individuals are being asked to do, the incentive for participating, and instructions on how to learn more information about engaging in the study. Interested individuals were also shown a 2-minute animated video describing the study, intervention, and eligibility to receive an incentive for completion of all intervention and survey components in addition to viewing the web-based consent form. Informed consent documentation was provided to participants at the beginning of the pretest survey and included details about the study aims, how it relates to the person accessing the consent form, what they would be asked to do, the risks and benefits, confidentiality of the data, the right to refuse or withdraw from the study, and a US $25 gift card incentive provided to the first 200 people completing the pretest, intervention, and posttest. All participants voluntarily consented to participate in the study. All data are anonymous, with no personally identifiable information.

## Results

The program resulted in significant changes in all 5 targeted psychosocial variables (Table 1). Knowledge of opioids, communicating with providers, pain management control, and intention to avoid opioid use all improved pretest-to-posttest. That is, participants knew more about opioids, understood the importance of communicating with providers about opioids and pain, improved their perception that they could appropriately control their pain, and intended to not use opioids inappropriately. Attitudes toward opioids were reversed in that lower scores reflected a less favorable attitude. Participants expressed more negative attitudes toward opioids at the posttest.

Additionally, the 33 participants who completed both the pretest and posttest provided positive ratings for the program (Table 2). They judged the program to present information that was appropriate, easy to understand, and engaging. They generally agreed that other people in their industry should participate in this program or one like it.

**Table 1:** Generalized repeated measures ANOVA mean changes in psychosocial outcome variables from pretest to posttest for 33 health care practitioners who completed the intervention and surveys (N=33).

	*F* test (*df*)	*P* value	Pretest mean	Posttest mean	Change
Knowledge of opioids	21.812 (1, 32)	<.001	3.524	3.983	0.459
Attitudes toward opioids	14.811 (1, 32)	.001	3.848	2.768	–1.081
Communicating with providers	7.609 (1, 32)	.01	5.174	5.379	0.205
Pain management control	16.991 (1, 32)	<.001	5.242	6.211	0.969
Intentions to avoid opioid use	5.060 (1, 32)	.03	5.795	6.008	0.212

**Table 2:** Descriptive statistics for posttest feedback about the program from 33 health care practitioners who completed the intervention and surveys (N=33).

	Strongly disagree, n (%)	Disagree, n (%)	Slightly disagree, n (%)	Slightly agree, n (%)	Agree, n (%)	Strongly agree, n (%)
The information presented in this program was too basic.	2 (6)	10 (30)	12 (36)	8 (24)	1 (3)	0 (0)
The information presented in this program was difficult to understand.	16 (49)	14 (42)	3 (9)	0 (0)	0 (0)	0 (0)
The information presented in this program was so boring that I became distracted.	6 (18)	19 (58)	6 (18)	2 (6)	0 (0)	0 (0)
All workers in my industry should complete a program like this.	1 (3)	1 (3)	1 (3)	7 (21)	13 (39)	10 (30)

## Discussion

### Overview

WorkWell is an mHealth opioid misuse prevention intervention designed to integrate with the lifestyle of workers with higher risks of opioid dispensing and misuse, such as those in the health care industry. The goal of this pilot study was to be a proof-of-concept test of the WorkWell intervention that focused on improving psychosocial factors that underlie preventing opioid use and misuse. Previous research [46] has demonstrated strong relationships between opioid intentions and the other psychosocial variables we tested. The 10-lesson intervention resulted in improvements in participants’ knowledge of opioids, attitudes toward opioids, communicating with providers, pain management control, and intentions to avoid opioid use. Furthermore, participants who completed all aspects—pretest, intervention, and posttest—reported favorably of the appropriateness, ease of use, and engagement of the intervention. However, 30% (14/47) of those who completed the pretest did not follow through with both intervention and posttest completion, biasing posttest results and missing the perspective of those who chose not to complete.

One major criticism of mHealth interventions is that participant use diminishes within a week or 2 of initiation if the information is static or relies on participants to initiate contact [54]. A hypothesized design strength of WorkWell is an intentional, scheduled approach to user engagement and retention, providing interactions and nudges based on participant response. However, questions remain about the effects of this design, as 30% (14/47) of the sample did not complete the full intervention and posttest. Although posttest data from health care workers who did complete WorkWell as a program generally agreed that it should be adopted as an industry standard for safety, these data exclude an important population of participants who chose not to complete the intervention and posttest. Whether these individuals dropped out due to the intervention or to COVID-19 stressors or workload, posttest results leave questions about the broader perceptions and value of this intervention. Thus, more research is needed to determine the effects of the intervention content and design on participants’ engagement and retention.

Similar to health care workers [48,49], construction workers also share the high rates of injury that put them at risk of getting prescription opioids to manage pain [55]. Workers in the construction industry have the highest rates of opioid misuse and opioid-related workplace mortality [47,56], suggesting a significant need and opportunity for an intervention that addresses opioid misuse at the primary prevention level. Future research is needed to examine whether the effects of the intervention remain significant across other at-risk populations, such as the construction industry.

Future research may also benefit from an examination of each intervention component. With interventions for workers, efficiency is imperative—though not at the cost of effectiveness. Although this intervention was developed with the intention of keeping all lessons brief and easily accessible, it is possible that the effects of the intervention are possible with fewer intervention components. We plan to use the multiphase optimization strategy (MOST) approach [57-60] to examine the effects of all intervention component combinations using a factorial design and control group in hopes of finalizing an effective and efficient intervention that can be used as a primary prevention method for opioid misuse among at-risk worker populations.

### Limitations

There are several limitations to this pilot study. Results are based on a small sample size of only 33 participants who completed the pretest, intervention, and posttest. The posttest results leave out the perspectives of the 14 individuals who dropped out of the study before completing the posttest, further biasing the overall findings for the effects of the intervention as well as the participant feedback. The pilot testing took place during the COVID-19 pandemic, creating challenges for recruiting and retaining the targeted population of health care workers who experienced higher workloads and strain during the pandemic. We did not capture data that would inform whether COVID-19 was a part of the reason for choosing not to participate in the full pilot study. We also did not include a control group and are unable to conclude whether increases in psychosocial variables would have been present without exposure to the intervention. Also, this pilot study included only one immediate posttest, limiting the ability to determine whether the intervention has lasting effects on intentions to avoid opioid misuse. Lastly, the intervention prototype was tested only with health care workers and is thus not generalizable to at-risk workers in other industries.

### Conclusions

While attempts to address the opioid crisis have been made through public health policy initiatives, the problem still permeates throughout our society as we can see opioid overdose deaths continue to rise. Prescription opioid misuse prevention efforts have focused heavily on restricting access to prescription opioids with drug monitoring programs, stricter prescribing guidelines, dose-limit laws, prescription take-back days, and increased law enforcement [61-64]. Interventions like WorkWell that focus on changing the individual and interpersonal motivational factors linked to opioid misuse could be an effective way to supplement current policy-level initiatives, though more research is needed to substantiate the effects of this particular program. Combining individual-level interventions with organizational or policy-level interventions provides a socioecological approach to preventing opioid misuse and overdose death. Additionally, because certain industries carry a greater risk for employee opioid use and misuse due to high rates of on-the-job injury [35,36], there is a specific need to provide easily accessible and scalable interventions for employees within these at-risk populations. This study presented an mHealth intervention that aims to mitigate opioid misuse and subsequent opioid morbidity and mortality among employees within an at-risk industry such as health care. Preliminary findings will inform the next phase of intervention development and testing, which will examine the effects of all combinations of intervention components against a control group to establish the most efficient intervention for use with at-risk workers using a MOST design [57-60].

## Abbreviations

MOST: multiphase optimization strategy

## Data Availability

The data set generated during this study is available from the corresponding author on reasonable request.
